# The viral hypothesis: how herpesviruses may contribute to Alzheimer’s disease

**DOI:** 10.1038/s41380-021-01138-6

**Published:** 2021-05-10

**Authors:** Michael Wainberg, Tain Luquez, David M. Koelle, Ben Readhead, Christine Johnston, Martin Darvas, Cory C. Funk

**Affiliations:** 1 Institute for Systems Biology, Seattle, WA USA; 2 Department of Medicine, University of Washington, Seattle, WA USA; 3 Vaccine and Infectious Disease Division, Fred Hutchinson Cancer Research Center, Seattle, WA USA; 4 Department of Global Health, University of Washington, Seattle, WA USA; 5 Benaroya Research Institute, Seattle, WA USA; 6 Department of Laboratory Medicine and Pathology, University of Washington, Seattle, WA USA; 7 Arizona State University-Banner Neurodegenerative Disease Research Center, Arizona State University, Tempe, AZ USA

**Keywords:** Molecular biology, Neuroscience

## Abstract

The hypothesis that infectious agents, particularly herpesviruses, contribute to Alzheimer’s disease (AD) pathogenesis has been investigated for decades but has long engendered controversy. In the past 3 years, several studies in mouse models, human tissue models, and population cohorts have reignited interest in this hypothesis. Collectively, these studies suggest that many of the hallmarks of AD, like amyloid beta production and neuroinflammation, can arise as a protective response to acute infection that becomes maladaptive in the case of chronic infection. We place this work in its historical context and explore its etiological implications.

## Background

Nine herpesviruses infect humans, all of which establish lifelong latency after infection. It has long been known that several herpesviruses, including herpes simplex virus-1 (HSV-1), establish latency in the peripheral nervous system. However, it was only with the advent of polymerase chain reaction (PCR) amplification that researchers discovered, in 1991, that trace amounts of HSV-1 DNA were also present in the brains of both AD patients and healthy controls [1], a finding since extended to several other herpesviruses. While the significance of this discovery was initially unclear, more recent studies from 2007 to 2014 identified multiple links between herpesviral brain infection and pathological hallmarks of AD (Table 1).

**Table 1:** Selected studies consistent with a contributory role of herpesviral infections to AD.

Study	Conclusions
Latent herpes simplex virus type 1 in normal and Alzheimer’s disease brains (Jamieson et al., J Med Virol. 1991 [1])	HSV-1 DNA is present in the brains of the majority of both normal and AD patients.
Herpes simplex virus infection causes cellular β-amyloid accumulation and secretase upregulation (Wozniak et al., Neurosci Lett. 2007 [2])	HSV-1 induces Aβ production and secretion in cultured neuronal and glial cells and wild-type mice by upregulating the Aβ-producing enzymes β-secretase 1 and γ-secretase.
Neuronal cytoskeletal dynamic modification and neurodegeneration induced by infection with herpes simplex virus type 1 (Zambrano et al., J Alzheimers Dis. 2008 [4])	HSV-1 infection causes tau hyperphosphorylation in mouse neuronal cultures.
Herpes simplex virus type 1 DNA is located within Alzheimer’s disease amyloid plaques (Wozniak et al., J Pathol. 2009 [3])	HSV-1 DNA is present in the core of 90% of Aβ plaques in postmortem brains from AD patients, and 72% of HSV-1 DNA in the brain was found within these plaques.
β-Amyloid peptides display protective activity against the human Alzheimer’s disease-associated herpes simplex virus-1 (Bourgade et al., Biogerontology. 2015 [5])	Aβ inhibits HSV-1 replication and viral entry in cell cultures.
Alzheimer’s disease-associated β-amyloid is rapidly seeded by Herpesviridae to protect against brain infection (Eimer et al., Neuron. 2018 [7])	Amyloid-β plaques form to entrap viruses in 5xFAD mice and are protective against herpes simplex encephalitis in 3D human neural cells.
Multiscale analysis of three independent Alzheimer’s cohorts reveals disruption of molecular, genetic, and clinical networks by Human herpesvirus (Readhead et al., Neuron. 2018 [9])	Increased HSV-1, HHV-6A, and HHV-7 viral RNA levels detected in postmortem brain samples of AD patients relative to controls. Herpesviruses predicted to modulate the expression of known AD-related genes.
The innate immunity protein IFITM3 modulates γ-secretase in Alzheimer’s disease (Hur et al., Nature. 2020 [15])	Interferon-γ treatment induced Aβ production in mouse neural cultures. IFITM3, a target of interferon signaling, physically interacts with γ-secretase and IFITM3 knockout impairs Aβ production in 5xFAD mice.
A 3D human brain-like tissue model of herpes-induced Alzheimer’s disease (Cairns et al., Sci Adv. 2020 [13])	HSV-1 infection of a 3D brain tissue model led to the formation of Aβ plaque-like structures, tau hyperphosphorylation, astrogliosis, neuroinflammation, and disrupted neuronal electrical activity. The antiherpetic drug valacyclovir prevented these changes.
Interaction between *APOE4* and herpes simplex virus type 1 in Alzheimer’s disease (Linard et al., Alzheimers Dement. 2020 [21])	*APOE4* carriers with recent HSV-1 reactivation (IgG+ and IgM+, or elevated IgG) developed AD at about 3× the rate of those merely infected with HSV-1 (IgG+); *APOE4* noncarriers showed no such association. This indicates a gene-by-environment interaction between *APOE4* and HSV-1 reactivation on AD risk.

Early efforts aimed to identify a relationship between herpes infection and amyloid beta (Aβ). Wozniak et al. [2] reported in 2007 that HSV-1 directly induced intracellular and extracellular Aβ production in cultured neuronal, glial cells and in wild-type mice by upregulating the Aβ-producing enzymes β-secretase 1 and γ-secretase. In 2009, Wozniak et al. [3] extended this observation by showing that HSV-1 DNA was present in 90% of Aβ plaques in postmortem brains from AD patients, and that 72% of HSV-1 DNA in the brain was found within these plaques. In 2008, a study by Zambrano et al. [4] found that HSV-1 infection of mouse neuronal cultures caused another hallmark of AD, tau hyperphosphorylation. In 2014, Bourgade et al. [5] demonstrated that Aβ peptides could inhibit HSV-1 replication and viral entry. This mirrored previous observations where Aβ was shown to have broad-spectrum antibacterial and antifungal activity [6]. Despite this evidence, the mechanistic connections between viral infection, AD genetic risk factors like *APOE4*, and AD etiology remained unclear.

## Main body

In 2018, Eimer et al. [7] reported two observations supporting a protective role for Aβ against herpesviruses. They found that 5xFAD mice (which overexpress variants of human *APP* and *PSEN1* containing five AD-linked causative mutations) survived encephalitis from intracranial HSV-1 injection better than wild-type mice. They also found that Aβ binds HSV-1 and HHV-6 surface glycoproteins—consistent with Cribbs et al. and Bourgade et al.’s observations that Aβ has sequence homology with HSV-1 glycoprotein B [5, 8]—and forms clumps that entrap viral particles. The authors hypothesized that Aβ deposition might protect against acute infection in the short term, but contribute to plaque formation over the long term in the case of chronic, low-grade infection.

Also in 2018, Readhead et al. [9] analyzed postmortem brain RNA-seq data from three cohorts and found evidence that herpesviruses modulate the expression of AD-related genes in AD patients’ brains. First, they found that RNA levels (a proxy for viral abundance) of three herpesviruses, HSV-1, HHV-6A, and HHV-7, were consistently increased in AD cases relative to controls. Second, they found genetic variants associated with viral RNA levels and used them to predict which viruses might alter the expression of host genes, and vice versa. Three herpesviruses were predicted to perturb host gene expression across all surveyed brain regions: HSV-1, HSV-2, and HHV-6A. Strikingly, the host genes most consistently associated with these viruses included many genes linked to Aβ production and/or AD risk. For instance, HHV-6A was associated with *BACE1* (which encodes β-secretase 1) and *PSEN1* (which encodes a subunit of γ-secretase), as well as *BIN1*, *CLU*, and *PICALM*—genes associated with AD in genome-wide association studies (GWAS). The most-associated genes also included those involved in innate immunity and antiviral sensing. These results generated some controversy, with a subsequent study [10] noting that Readhead et al. also reported reads mapping to hepatitis C and variola (smallpox) virus in nearly 100% of brain samples from one American cohort—even though hepatitis C infects under 1% of Americans [11] and variola virus was eradicated in the 1970s—perhaps due to sequence homology with other viruses or genomically integrated transposable elements. Despite these false positives, neither hepatitis C nor variola virus emerged as AD-relevant species from the authors’ integrative analysis. Another analysis of the same cohorts [12] found similar HHV-6A and HHV-7 levels in AD cases and controls, though this negative result is difficult to interpret since this analysis only detected HHV-6A DNA or RNA in <1% of brain samples—over an order of magnitude less than both Readhead et al. and prior studies of other cohorts.

While many previous studies of herpesvirus infection and AD used primary culture or animal models, it remained unclear whether similar effects would be observed in more complex human tissue models. In 2020, Cairns et al. [13] approached this question with 3D brain organoids made of silk sponges infused with induced human neural stem cells, which spontaneously differentiate into a variety of neuronal and glial subtypes. HSV-1 infection of this human tissue model elicited AD hallmarks like Aβ plaque-like formations containing hyperphosphorylated tau, gliosis, neuroinflammation, decreased local field potential (indicative of disrupted neurotransmission), and neuronal death. Just like in a previous cell culture study [14], the antiherpetic drug valacyclovir largely rescued these changes, with early administration (same day as HSV-1 infection) more effective than later (1 day post-infection). Thus, HSV-1 infection was sufficient to induce AD-like pathophysiology in a human tissue model, and this was largely preventable by timely administration of an anti-herpes drug. This study provides perhaps the most compelling evidence to date for a contributory role of HSV-1 to AD.

Also in 2020, Hur et al. [15] reported that interferons, key signaling proteins of the innate immune system’s antiviral response, can induce Aβ production. Treatment with interferon-γ led to a ~70% increase in Aβ secretion from mouse primary neurons. They also showed that interferon-induced transmembrane protein 3 (IFITM3), a target of interferon signaling, physically interacts with γ-secretase and that *Ifitm3* knockout impairs Aβ production. Another 2020 study by Roy et al. [16] reported an interaction in the opposite direction, finding that Aβ fibrils containing nucleic acids (but not those without) could elicit a type I interferon response from mouse microglia. These studies suggest that herpesvirus-induced interferon signaling could accelerate Aβ deposition, and that viral nucleic acids entrapped by Aβ could trigger further interferon signaling, potentially leading to a positive feedback loop.

An emerging area of interest with respect to both herpesvirus infection and AD is the adaptive immune system. AD GWAS have implicated the HLA class II region, which is primarily involved in adaptive immunity. One potential mechanism that could mediate this association is herpesviral reactivation. Herpesviruses lie latent in the periphery and are kept in check by the adaptive immune system, so adaptive immune function could influence the frequency and/or intensity of viral reactivation and spread to the central nervous system (CNS). In 2020, Gate et al. [17] found greater clonal expansion of CD8+ T cells with epitope specificity for Epstein–Barr virus in the cerebrospinal fluid of AD patients compared to healthy controls, suggesting a greater adaptive immune response against this herpesvirus in the CNS. Future work is necessary to determine what etiological consequences, if any, this may have for AD.

Further supporting a potential role for herpesviral reactivation in AD pathophysiology, a 2019 study from De Chiara et al. [18] showed that wild-type mice infected with HSV-1 developed Aβ accumulation, tau hyperphosphorylation, and neuroinflammation after several cycles of reactivation with thermal stress (15 min in a 43 °C water bath) compared to controls exposed to the thermal stress but not infected with HSV-1. Seven reactivation cycles produced more of the pro-inflammatory cytokines IL-6 and IL-1β than three cycles. Mice subjected to more than seven reactivation cycles had irreversible cognitive deficits. A limitation of this study is that the authors did not demonstrate that the observed pathology was due to reactivation rather than initial infection.

Many population studies have found associations between AD and HSV-1 diagnosis [19] or reactivation [20]. For instance, a 2018 Taiwanese insurance claims study [19] reported that HSV diagnosis predicted a 2.5-fold higher rate of subsequent dementia diagnosis, but not among those prescribed antiherpetic drugs. However, these studies were purely epidemiological in nature and thus potentially subject to confounding. In 2020, Linard et al. [21] used a more robust gene-by-environment interaction study design to find that *APOE4* carriers with recent HSV-1 reactivation (IgG+ and IgM+, or elevated IgG) developed AD at ~3× the rate of those merely infected with HSV-1 (IgG+); *APOE4* noncarriers showed no such association. This suggests that *APOE4* may increase the harmful effects of HSV-1 reactivation on AD risk, perhaps by increasing brain viral titers as it does in mice [22]. This study builds on results from the late 1990s [23, 24] that relied on postmortem HSV-1 PCR positivity in brain instead of antemortem seropositivity, which had the disadvantages of very small sample size, non-longitudinal designs, and lack of consistent replication [25, 26]. It also builds on more recent studies showing gene-by-environment interactions between *APOE4* and HSV-1 seropositivity [27] and interactions between APOE genotype and HSV-1 neuroinvasiveness and latent viral load in mice [22]. A limitation of both Linard et al. and earlier gene-by-environment interaction studies is lack of correction for population structure (i.e., some pairs of individuals being more genetically similar to each other than others, even within a relatively homogeneous population), a common confounder in genetic association analyses [28].

## Conclusions

While herpesviruses have been argued to contribute to AD for decades, recent studies have rekindled the debate. Collectively, these studies suggest that (1) Aβ aggregation is a protective mechanism to entrap herpesviruses (and perhaps other pathogens), (2) herpesviruses trigger the main hallmarks of AD in human brain tissue models, (3) herpesvirus-induced interferon signaling can trigger Aβ production, and (4) *APOE4* increases the effect of herpesviral reactivation on AD risk. Strikingly, herpesviruses appear to be intimately related to both the largest genetic risk factor for AD (*APOE4*) and its most classic hallmarks (Aβ and tau).

The extent to which herpesviruses causally contribute to AD remains unclear. Certainly, herpesviruses are not the sole contributor: AD is a multifactorial disease with many contributory genetic and environmental factors (including, potentially, other pathogens [29]). Herpesviruses may only causally contribute to a minority of AD cases, and even then the effect size of this causal contribution may depend on the presence or absence of other risk factors.

Viewed another way, the causal effect size of these other risk factors may depend on the presence or absence of herpesviruses. Environmental factors like stress, diet, sleep, and exercise may influence AD risk in part by modulating the innate and adaptive immune responses to herpesviruses. As for genetic risk factors, the gene-by-environment interaction studies discussed here suggest that HSV-1 influences the amount of AD risk conferred by *APOE4*. The same may be true for other AD genetic factors like the protective G78R variant of *PILRA* [30], a co-receptor for HSV-1 entry [31]. A similar phenomenon has been observed in a mouse model of a Mendelian form of Parkinson’s: *Pink1* knockout mice did not develop Parkinson’s symptoms unless inoculated with Gram-negative bacteria [32]. If not for herpesviruses, certain AD GWAS hits might have smaller effect sizes, or even disappear.

It is worth reflecting on what future evidence might strengthen or refute the contributory hypothesis of herpesvirus infections in AD. So far, arguably the strongest evidence comes from perturbational studies showing that herpesvirus infection causes hallmarks of AD. Perturbational studies avoid the issues of confounding and reverse causality that plague associative or epidemiological studies, but have so far relied on animal and in vitro models. These models are imperfect representations of AD: for instance, animal models are often based on mutations in a single gene or disease pathway, even the best in vitro models fail to capture the structural intricacies of the human brain and its interactions with the periphery, and both types of models have difficulty capturing inter-individual variability and environmental contributors to AD risk. Fortunately, one sort of human perturbational study is ongoing: two randomized control trials of whether valacyclovir delays progression in HSV+ AD patients (NCT03282916 and NCT02997982 at ClinicalTrials.gov). A positive result from these trials would further strengthen the evidence for the contributory hypothesis as well as confirming its clinical relevance. However, a negative result would not necessarily refute the hypothesis: by the time individuals develop symptomatic AD, antiherpetics may already be “too little too late” [33]. Clinical trials of whether antiherpetics can reduce AD incidence among HSV-1-seropositive individuals with mild cognitive impairment—and particularly those who also have the *APOE4* allele—might provide a more definitive answer [34].

Collectively, the studies summarized here provide interlocking evidence for a contributory role for herpesviruses to AD, in conjunction with established etiological factors. Fundamentally, they support the model articulated by Eimer et al. in which the host’s rational response to acute infection or reactivation—Aβ production for viral entrapment and neuroinflammation for viral clearance—becomes maladaptive in the case of chronic infection.
